# Risk scores for choledocholithiasis perform poorly in patients with hemolytic diseases: a PEDI database report

**DOI:** 10.3389/fped.2025.1574462

**Published:** 2025-04-08

**Authors:** Jennifer Thompson, Wenly Ruan, Douglas S. Fishman, Matthew Giefer, Kyung Mo Kim, Mercedes Martinez, Luigi Dall'Oglio, Valerio Balassone, Filippo Torroni, Paola De Angelis, Simona Faraci, Cynthia Tsai, Michael Wilsey, Racha Khalaf, Petar Mamula, Quin Liu, Yuhua Zheng, Bradley A. Barth, David Michael Troendle

**Affiliations:** ^1^ Department of Pediatrics, Baylor College of Medicine, Houston, TX, United States; ^2^ Department of Pediatrics, Ochsner Hospital for Children, New Orleans, LA, United States; ^3^ College of Medicine, University of Ulsan, Ulsan, Republic of Korea; ^4^ Department of Pediatrics, Columbia University, New York City, NY, United States; ^5^ Digestive Endoscopy and Surgery Unit, Bambino Gesù Children’s Hospital (IRCCS), Rome, Italy; ^6^ Department of Pediatrics, Johns Hopkins All Children’s Hospital, Saint Petersburg, FL, United States; ^7^ Department of Pediatrics, University of South Florida, Tampa, FL, United States; ^8^ Department of Pediatrics, Children’s Hospital of Philadelphia, Philadelphia, PA, United States; ^9^ Department of Pediatrics, Cedars Sinai Medical Center, Los Angeles, CA, United States; ^10^ Department of Pediatrics, University of Southern California, Los Angeles, CA, United States; ^11^ Department of Pediatrics, University of Texas Southwestern Medical Center, Dallas, TX, United States; ^12^ Department of Pediatrics, Children’s Health, Children’s Medical Center Dallas, Dallas, TX, United States

**Keywords:** ERCP, choledocholithiasis, hemolytic disease, sickle cell, predictor

## Abstract

Patients with hemolytic diseases are at increased risk for gallstone-related complications. Modified scoring systems have been developed to assess which pediatric patients would benefit from endoscopic retrograde cholangiopancreatography (ERCP) to treat choledocholithiasis. This study aimed to evaluate the ability of the available criteria to determine which pediatric patients with hemolytic diseases are likely to benefit from ERCP. A secondary analysis was performed using the Pediatric ERCP Database Initiative database, which contains prospectively collected data from 1,124 ERCPs at tertiary-care institutions. We compared patients with a hemolytic disease to those without. Data was analyzed by two-tailed Fisher’s exact test and paired student *t*-test. Of the 47 (17.0%) patients who had a hemolytic disease, 34 (72.3%) had one or more common bile duct (CBD) stones at the time of ERCP. Among patients with hemolytic diseases, there were no differences in pre-ERCP imaging or laboratory findings between those with a CBD stone removed at ERCP and those without. Patients with hemolytic diseases did not fit the current choledocholithiasis selection criteria well: 80% in the no-stone at ERCP group met the American Society of Gastrointestinal Endoscopy high-risk criteria, and 90% met the 2016 modified Baylor pediatric criteria. Although not statistically significant, there was an increased number of adverse events in patients with hemolytic diseases. Existing ERCP criteria perform poorly in patients with hemolytic diseases, overestimating their risk of choledocholithiasis. Peri-procedure evaluations such as endoscopic ultrasound, magnetic resonance cholangiopancreatography, and intraoperative cholangiography appear underutilized and may be essential modalities in this population.

## Introduction

Pediatric patients with hemolytic diseases, including sickle cell disease, thalassemia, and red cell membrane disorders like hereditary spherocytosis, are at increased risk for gallstone-related complications such as choledocholithiasis (1, 2). However, risk stratification for patients who would benefit from endoscopic retrograde cholangiopancreatography (ERCP) in this population is less straightforward than the general pediatric population given baseline elevations in bilirubin and transaminases in the setting of ongoing hemolysis. Additionally, the role of intrahepatic sickling, with or without infarction, in sickle cell disease is often difficult to assess when there is co-existing gallbladder disease. Chronic liver injury secondary to iron overload can also contribute to baseline laboratory abnormalities in patients who receive frequent blood transfusions (2).

The ability to accurately predict choledocholithiasis using non-invasive measures such as laboratory markers and imaging is of great interest to avoid non-therapeutic ERCP and its associated risks and costs (3). Non-therapeutic ERCP refers to ERCP in which a gallstone is not identified in the common bile duct during the procedure. Recent guidelines have moved to increase specificity in the parameters to minimize these “negative” ERCPs (3). Modified pediatric scoring systems have been developed to assess which patients would benefit from ERCP. These scoring systems are primarily based on bilirubin and common bile duct (CBD) diameter (4–6). Specifically, conjugated bilirubin has been found to have a higher sensitivity than total bilirubin in predicting choledocholithiasis in pediatric patients (5). With this in mind, the primary aim of this study was to evaluate the ability of the currently recommended criteria to determine which pediatric patients with hemolytic diseases would benefit from ERCP to treat choledocholithiasis.

## Methods

A secondary analysis was performed using the Pediatric ERCP Database Initiative (PEDI) database. This international database contains prospectively collected data from 1,124 ERCPs performed at 13 large, tertiary-care institutions. This study was part of a distinct substudy involving 8 of the 13 centers. Inclusion criteria were age less than 19 years and ERCP performed for suspected choledocholithiasis. Patients were excluded if they had a prior ERCP or intervention for choledocholithiasis, such as laparoscopic common bile duct exploration (LCBDE). Detailed information on this cohort has been published (7). The study was approved by each individual center's institutional review board, and informed consent was obtained as per each institution's institutional review board requirements.

Information was collected on patient demographics [e.g., age, sex, race, and body mass index (BMI)] and clinical presentation, including right upper quadrant pain at rest, right upper quadrant pain with palpation, jaundice, and nausea. It was also noted whether the patient had a diagnosis of gallstone pancreatitis or cholangitis. For patients over two years of age, obesity was defined as BMI greater than or equal to the 95th percentile for age and sex.

Patients' pre-ERCP imaging findings were assessed for the presence of a common bile duct stone. Imaging techniques used at the various institutions included ultrasound, magnetic resonance cholangiopancreatography (MRCP), intraoperative cholangiography (IOC), and endoscopic ultrasound (EUS). The presence of a common bile duct stone on intraoperative cholangiography was defined as no duodenal filling of contrast with filling of the CBD or identification of a fixed or mobile filling defect in the CBD. In the other imaging modalities, the presence of a common bile duct stone was identified visually, by shadowing on the sonograms or a filling defect on MRCP. Laboratory indices were also collected, including total bilirubin, conjugated or direct bilirubin, alanine aminotransferase, aspartate aminotransferase, gamma-glutamyl transferase, and alkaline phosphatase, at the time of admission and within 24 h of ERCP. ERCP outcomes were defined as whether a stone was identified and whether it was removed from the common bile duct during ERCP. Adverse events were categorized using the American Society for Gastrointestinal Endoscopy (ASGE) lexicon as pancreatitis, bleeding, failed cannulation, or other adverse events (8).

Patient data was evaluated in relation to the 2019 ASGE high-risk criteria, which include common bile duct diameter greater than 6 mm, total bilirubin greater than 4 mg/dl, clinical ascending cholangitis, or stone seen on ultrasound or cross-sectional imaging (3), and the 2016 modified Baylor pediatric criteria, which include common bile duct diameter greater than 6 mm and conjugated bilirubin greater than or equal to 0.5 mg/dl (5).

Study data were collected and managed using Research Electronic Data Capture (REDCap Software, Vanderbilt University) tool hosted at University of Texas Southwestern Medical Center. REDCap is a secure, web-based application designed to support data capture for research studies, providing (1) an intuitive interface for validated data entry, (2) audit trails for tracking data manipulation and export procedures, (3) automated export procedures for seamless data downloads to common statistical packages, and (4) methods for importing data from external sources. This study was supported by the UT Southwestern Academic Information System (CTSA NIH Grant UL1-RR024982) and the REDCap project. Data was analyzed by two-tailed Fisher's exact test and paired student *t*-test, with statistical significance drawn at *p* < 0.05.

## Results

296 patients who required ERCP were enrolled from 8 of the 13 sites within the PEDI registry. Of those, 11 patients without hemolytic diseases were excluded for prior ERCP, and 8 patients with hemolytic diseases were excluded for prior ERCP or LCBDE. Of the 277 patients meeting inclusion criteria, 47 (17.0%) had a hemolytic condition.

Demographics differed between the patients with hemolytic diseases and those without. Patients with hemolytic diseases were more likely to be of African American race (42.6% vs. 10.0%, *p* = 0.0001). Patients without hemolytic diseases were more likely to be of Hispanic ethnicity (60.0% vs. 17.0%, *p* = 0.0001), female sex (80.0% vs. 44.7%, *p* = 0.0001), and to have obesity (40.2% vs. 10.9%, *p* = 0.0001) (Table 1a).

**Table 1:** Demographics and clinical features of patients with and without hemolytic diseases who received ERCP.

Variable	Total (*n* = 277)	Patients with hemolytic diseases (*n* = 47)	Patients without hemolytic diseases (*n* = 230)	*P*-value
1a) Patient demographics				
Age in years	14.7 [12.4–16.3]	13.5 [10.6–15.7]	14.9 [12.7–16.3]	0.61
Female	205 (74.0%)	21 (44.7%)	184 (80.0%)	0.0001^b^
Hispanic	146 (52.7%)	8 (17.0%)	138 (60.0%)	0.0001^b^
White	213 (76.9%)	24 (51.1%)	189 (82.2%)	0.0001^b^
African American	43 (15.5%)	20 (42.6%)	23 (10.0%)	0.0001^b^
Obesity^a^	91 (35.0%)^a^	5 (10.9%)^a^	86 (40.2%)^a^	0.0001^b^
1b) Clinical features				
Gallstone pancreatitis	65 (23.5%)	7 (14.9%)	58 (25.2%)	0.18
Cholangitis	8 (2.9%)	2 (4.3%)	6 (2.6%)	0.63
RUQ pain at rest	242 (87.4%)	39 (83.0%)	203 (88.3%)	0.34
RUQ pain on palpation	209 (75.5%)	31 (66.0%)	178 (77.4%)	0.84
Jaundice	109 (39.4%)	37 (78.7%)	72 (31.3%)	0.0001^b^
Nausea	195 (70.4%)	24 (51.1%)	171 (74.3%)	0.0026^b^

^a^ Excludes 17 from total: 1 from hemolytic group, 16 from nonhemolytic group, for missing data or age less than 2 years.

^b^ Significant by two tailed Fisher's exact test.

Clinically, patients with hemolytic diseases were more likely to have jaundice (78.7% vs. 31.3%, *p* = 0.0001) and less likely to have nausea (51.5% vs. 74.3%, *p* = 0.0026) than their non-hemolytic counterparts. There was no difference in right upper quadrant pain at rest (83.0% vs. 88.3%, *p* = 0.34) or with palpation (66.0% vs. 77.4%, *p* = 0.84) between the two groups. The incidence of gallstone pancreatitis (14.9% vs. 25.2%, *p* = 0.18) and cholangitis (4.3% vs. 2.6%, *p* = 0.63) did not differ between the two groups (Table 1b).

Among the 47 patients with hemolytic diseases, 34 (72.3%) had a CBD stone identified at the time of ERCP. The remaining 13 patients underwent ERCP but did not have a stone in the common bile duct at the time of the procedure, resulting in non-therapeutic ERCP. This is similar to previously published data on patients without hemolytic diseases from the PEDI Database, where 69 out of 95 (72.6%) had a CBD stone removed at ERCP (4). Among patients with hemolytic diseases, those with a stone removed at ERCP were more likely to present with jaundice (82.4% vs. 46.2%, *p* = 0.03). There was no difference in age, race, ethnicity, sex, obesity, gallstone pancreatitis, or cholangitis between the patients with hemolytic diseases who had a stone removed at ERCP and those who did not (Tables 2a,b). There was also no significant difference in pre-ERCP laboratory or imaging findings, including common bile duct stone on ultrasound, MRCP, intraoperative cholangiography, or endoscopic ultrasound (Tables 3a,b). However, the number of patients who received endoscopic ultrasound or intraoperative cholangiography was low (*n* = 6 and 1, respectively).

**Table 2:** Demographics and clinical features of patients with hemolytic diseases who received ERCP.

Variable	Total (*n* = 47)	CBD stone removed at ERCP (*n* = 34)	No CBD stone removed at ERCP (*n* = 13)	*P*-value
2a) Patient demographics				
Age in years	13.5 [8.6–15.7]	13.0 [10.5–15.6]	14.3 [12.1–15.5]	0.53
Female	21 (44.7%)	14 (41.2%)	7 (53.8%)	0.52
Hispanic	8 (17.0%)	5 (14.7%)	1 (7.7%)	1.00
White	22 (46.8%)	12 (35.3%)	8 (61.5%)	0.19
African American	20 (42.6%)	15 (44.1%)	4 (30.8%)	0.51
Obesity^a^	5 (10.8%)^a^	5 (15.2%)^a^	0 (0.0%)	0.30
2b) Clinical features				
Gallstone pancreatitis	7 (14.9%)	3 (8.8%)	4 (30.8%)	0.08
Cholangitis	2 (4.3%)	1 (2.9%)	1 (7.7%)	0.49
RUQ pain at rest	21 (44.7%)	16 (47.1%)	5 (38.5%)	0.74
RUQ pain on palpation	25 (53.2%)	16 (47.0%)	9 (69.2%)	0.21
Jaundice	34 (72.3%)	28 (82.4%)	6 (46.2%)	0.03^b^
Nausea	8 (17.0%)	6 (17.6%)	2 (15.4%)	1.00

^a^ Excludes 1 from the CBD stone removed at ERCP group due to age less than two years.

^b^ Significant by two tailed Fisher's exact test.

**Table 3:** Imaging findings and laboratory values of patients with hemolytic diseases who received ERCP.

Variable	Total^a^	CBD stone removed at ERCP	No CBD stone removed at ERCP	*P*-value
3a) Imaging findings				
CBD stone on US	14/42 (33.3%)	13/33 (39.4%)	1/9 (11.1%)	0.23
CBD stone on MRCP	6/9 (66.7%)	2/5 (40.0%)	4/4 (100%)	0.17
CBD stone on IOC	1/1 (100.0%)	1/1 (100.0%)	0/0 (0%)	1.00
CBD stone on EUS	3/6 (50.0%)	3/4 (75.0%)	0/2 (0%)	0.40
CBD stone on any pre-ERCP imaging	23/44 (52.3%)	18/34 (52.9%)	5/10 (50.0%)	1.00
3b) Laboratory values [mean, (IQR)]				
Total bilirubin (*n* = 42)	15.0 [6.5–21.9]	14.3 [6.6–21.7]	17.2 [4.5–27.3]	0.48
Direct or conjugated bilirubin (*n* = 42)	10.1 [1.8–15.4]	8.8 [2.1–15.1]	14.4 [1.1–27.2]	0.13
ALT (*n* = 41)	244 [121–322]	278 [131–365]	139 [58–230]	0.053
AST (*n* = 41)	174 [93–178]	198 [113–189]	87 [50–111]	0.08
GGT (*n* = 38)	226 [138–241]	208 [138–235]	285 [152–314]	0.21
Alkaline phosphatase (*n* = 39)	269 [157–346]	264 [143–338]	284 [182–388]	0.72
3c) Criterion assessment				
2019 ASGE high-risk criteria	34/42 (81.0%)	26/32 (81.3%)	8/10 (80.0%)	1.00
2016 modified baylor pediatric criteria	39/42 (92.9%)	30/32 (93.8%)	9/10 (90.0%)	1.00

^a^ The denominator in this column indicates the total number of patients who underwent each imaging modality.

Previously published selection criteria, including the ASGE high-risk criteria and the 2016 modified Baylor pediatric criteria, performed poorly in the patients with hemolytic diseases. Of the patients with hemolytic diseases who did not have a stone at the time of ERCP, 80.0% met the ASGE high-risk criteria, and 90.0% met the modified Baylor pediatric criteria. By comparison, 81.3% of patients with hemolytic diseases who had one or more stones removed at the time of ERCP met the ASGE high-risk criteria (*p* = 1) and 93.8% met the modified Baylor pediatric criteria (*p* = 1) (Table 3c).

While procedural technical success between patients with and without hemolytic diseases was similar (95.7% vs. 97.8%, *p* = 0.34), adverse events were experienced twice as commonly in the hemolytic population. However, this was not statistically significant (12.8% vs. 5.2%, *p* = 0.09). Specifically, the difference in bleeding between the two groups approached significance, with 6.4% of the patients with hemolytic diseases and 1.7% of those without hemolytic diseases experiencing bleeding after ERCP (*p* = 0.10) (Table 4).

**Table 4:** Adverse events and technical outcomes among pediatric patients with and without hemolytic diseases who received ERCP.

Adverse event	Total (*n* = 277)	Patients with hemolytic diseases (*n* = 47)	Patients without hemolytic diseases (*n* = 230)	*P*-value
Any adverse event	18 (6.5%)	6 (12.8%)	12 (5.2%)	0.09
Pancreatitis	11 (4.0%)	2 (4.3%)	9 (3.9%)	1.00
Bleeding	7 (2.5%)	3 (6.4%)	4 (1.7%)	0.10
Stone removed at ERCP	194 (70.0%)	34 (72.3%)	160 (69.6%)	0.86
Failed cannulation	7 (2.5%)	2 (4.3%)	5 (2.2%)	0.34

## Discussion

This is the first pediatric study to specifically evaluate patients undergoing ERCPs with underlying hemolytic disorders. We found that existing ERCP criteria perform poorly in patients with hemolytic diseases. The existing criteria overestimate their risk of choledocholithiasis, possibly resulting in unnecessary ERCPs and their associated risks and costs. Current criteria rely on pre-procedure laboratory and imaging findings; in our cohort, there were no differences in these pre-ERCP evaluations in patients with hemolytic diseases with a common bile duct stone removed at ERCP and those without. These patients' clinical presentations and laboratory values, especially bilirubin, can be clouded by disease-specific factors such as ongoing hemolysis, intrahepatic sickling with or without infarction (in sickle cell disease), and iron overload related to blood transfusions. Total bilirubin includes components of conjugated or direct and unconjugated or indirect bilirubin. Unconjugated bilirubin in the setting of hemolysis is expected to be elevated. Therefore, risk stratification for choledocholithiasis is more complicated than in the general pediatric population and continues to be challenging. The most recent ASGE guideline made a conscious effort to increase specificity to greater than 90% by using more stringent criteria (3).

The poor performance of bile duct size and identification of a stone on ultrasound is consistent with non-hemolytic pediatric data that our group has previously published, in which there was no significant difference in ultrasound findings between patients who had a stone removed during ERCP and those who did not (4). Ultrasound overall seems to have poor sensitivity in detecting choledocholithiasis but is frequently used given its accessibility and lack of radiation.

Few patients in our study underwent other peri-procedure evaluations such as endoscopic ultrasound, MRCP, or intraoperative cholangiography, as the use and timing of these modalities is center-dependent. Nevertheless, these may be very important modalities given the poor ability to predict choledocholithiasis through labs and other imaging modalities in patients with hemolytic diseases. For example, a prior study demonstrated that MRCP better predicts choledocholithiasis in children than transabdominal ultrasound or CT, though it is important to consider the timing of MRCP in relation to ERCP as stones may pass if there is delay (4). The delay between MRCP and ERCP is of particular relevance in pediatrics, as many pediatric patients require anesthesia to undergo MRCP and therefore MRCP is oftentimes not performed on the same day as the ERCP, potentially allowing time for the stone to pass prior to ERCP. Additionally, some pediatric centers lack the ability to perform sedated MRCP on weekends.

While very few children in our study underwent intraoperative cholangiogram (IOC) or endoscopic ultrasound (EUS) (*n* = 1 and 6, respectively), these modalities appeared to perform well; the patient who had a positive IOC had a stone removed during ERCP, and all three of the patients who had a positive EUS had a stone removed during ERCP (Table 3). However, these are very small samples. EUS has been recommended in select patients to diagnose choledocholithiasis with good specificity (9, 10). However, in the adult literature, EUS is thought to have higher sensitivity for detecting small stones (9, 10). While EUS is a relatively recent development in pediatric gastroenterology, it has been shown to be safe and have good technical outcomes in children (11–13). EUS is radiation-sparing and, in at least two studies in the pediatric population, has been shown to successfully prevent a subset of patients from undergoing non-therapeutic ERCP (12, 13). Regarding IOC, there is some data that performing IOC prior to planned ERCP can prevent non-therapeutic ERCP in a subset of patients who have preoperative labs and imaging that are falsely concerning for choledocholithiasis (14).

Overall, increased use of peri-procedure evaluations may decrease the incidence of non-therapeutic ERCPs in the hemolytic disease population, but more data is needed to determine acceptable sensitivity and specificity of these modalities in pediatric patients. Additionally, the potential costs and risk of additional time spent under sedation to perform these evaluations must be weighed clinically. This dilemma is of particular relevance in the hemolytic disease population. For example, when considering patients with sickle cell disease who have a history of stroke or acute chest syndrome, one must balance the desire to avoid anesthesia exposure with potential ERCP complications such as pancreatitis, which could also prompt an episode of acute chest syndrome.

Finally, while not statistically significant, the increased number of adverse events in patients with hemolytic diseases highlights the importance of careful patient selection for ERCP. In particular, the suggestion of increased bleeding in patients with hemolytic diseases is of interest. Our incidence of bleeding in 2.5% of patients (7/277) is slightly higher than the previously published incidence of 1.2% from the PEDI database, though we did not sub-categorize by severity of bleeding in this substudy and the difference between patients with and without hemolytic diseases was not significant (7). Sickle cell disease, the most common hemolytic disease in our patient population, is typically conceptualized as a prothrombotic state that predisposes patients to cerebral infarction and venous thromboembolism (15); however, there are some reports of spontaneous bleeding events in adults with sickle cell disease, mainly in the gastrointestinal tract (16). A plausible contributing factor to bleeding is heavy NSAID use due to chronic sickle cell-related pain, but this is unlikely to fully explain the bleeding risk in this population. Overall, further investigation is needed to determine which factors would better predict the need for ERCP in patients with hemolytic diseases.

## Data Availability

The datasets presented in this article are not readily available because data release is restricted by data use agreements between each of the participating sites and UT Southwestern Medical Center. Requests to access the datasets should be directed to david.troendle@utsouthwestern.edu.

## References

[1] Mohamed SOO Ibrahim OAO Mohammad DAA Ali AHM. Correlates of gallbladder stones among patients with sickle cell disease: a meta-analysis. JGH Open. (2021) 5:997–1003. 10.1002/jgh3.12622 34584966 PMC8454478

[2] Ebert EC Nagar M Hagspiel KD. Gastrointestinal and hepatic complications of sickle cell disease. Clin Gastroenterol H. (2010) 8:483–9. 10.1016/j.cgh.2010.02.016 20215064

[3] Committee AS of P, Buxbaum JL Fehmi SMA Sultan S Fishman DS Qumseya BJ ASGE Guideline on the role of endoscopy in the evaluation and management of choledocholithiasis. Gastrointest Endosc. (2019) 89:1075–105.e15. 10.1016/j.gie.2018.10.001 30979521 PMC8594622

[4] Fishman DS Barth B Tsai CM-W Giefer MJ Martinez M Wilsey M A prospective multicenter analysis from the pediatric ERCP database initiative: predictors of choledocholithiasis at ERCP in pediatric patients. Gastrointest Endosc. (2021) 94:311–17.e1. 10.1016/j.gie.2021.01.030 33539907

[5] Fishman DS Chumpitazi BP Raijman I Tsai CM-W Smith EO Mazziotti MV Endoscopic retrograde cholangiography for pediatric choledocholithiasis: assessing the need for endoscopic intervention. World J Gastrointest Endosc. (2016) 8:425–32. 10.4253/wjge.v8.i11.425 27298714 PMC4896904

[6] Capparelli MA D'alessandro PD Questa HA Ayarzabal VH Bailez MM Barrenechea ME. Development of a risk score for choledocholithiasis in pediatric patients. Pediatr Surg Int. (2021) 37:1393–9. 10.1007/s00383-021-04952-9 34146133

[7] Troendle DM Ruan W Fishman DS Barth BA Liu QY Giefer M Technical outcomes in pediatric endoscopic retrograde cholangiopancreatography. J Pediatr Gastroenterol Nutr. (2022) 75:755–60. 10.1097/mpg.0000000000003612 36122368

[8] Cotton PB Eisen GM Aabakken L Baron TH Hutter MM Jacobson BC A lexicon for endoscopic adverse events: report of an ASGE workshop. Gastrointest Endosc. (2010) 71:446–54. 10.1016/j.gie.2009.10.027 20189503

[9] Manes G Paspatis G Aabakken L Anderloni A Arvanitakis M Ah-Soune P Endoscopic management of common bile duct stones: European society of gastrointestinal endoscopy (ESGE) guideline. Endoscopy. (2019) 51:472–91. 10.1055/a-0862-0346 30943551

[10] Meeralam Y Al-Shammari K Yaghoobi M. Diagnostic accuracy of EUS compared with MRCP in detecting choledocholithiasis: a meta-analysis of diagnostic test accuracy in head-to-head studies. Gastrointest Endosc. (2017) 86:986–93. 10.1016/j.gie.2017.06.009 28645544

[11] Mahajan R Simon EG Chacko A Reddy DV Kalyan PR Joseph AJ Endoscopic ultrasonography in pediatric patients—experience from a tertiary care center in India. Indian J Gastroenterol. (2016) 35:14–9. 10.1007/s12664-016-0619-2 26946134

[12] Barakat MT Cagil Y Gugig R. Landscape of pediatric endoscopic ultrasound in a United States tertiary care medical center. J Pediatr Gastroenterol Nutr. (2022) 74:657–61. 10.1097/mpg.0000000000003403 35149652

[13] Scheers I Ergun M Aouattah T Piessevaux H Borbath I Stephenne X Diagnostic and therapeutic roles of endoscopic ultrasound in pediatric pancreaticobiliary disorders. J Pediatr Gastroenterol Nutr. (2015) 61:238–47. 10.1097/mpg.0000000000000692 25564818

[14] Waldhausen JHT Graham DD Tapper D. Routine intraoperative cholangiography during laparoscopic cholecystectomy minimizes unnecessary endoscopic retrograde cholangiopancreatography in children. J Pediatr Surg. (2001) 36:881–4. 10.1053/jpsu.2001.23960 11381417

[15] Shet AS Lizarralde-Iragorri MA Naik RP. The molecular basis for the prothrombotic state in sickle cell disease. Haematologica. (2020) 105:2368–79. 10.3324/haematol.2019.239350 33054077 PMC7556662

[16] Hariharan N Brunson A Mahajan A Keegan THM Wun T. Bleeding in patients with sickle cell disease: a population-based study. Blood Adv. (2020) 4:793–802. 10.1182/bloodadvances.2019000940 32108229 PMC7065478

